# Adolescent Intermittent Ethanol Drives Modest Neuroinflammation but Does Not Escalate Drinking in Male Rats

**DOI:** 10.3390/cells12212572

**Published:** 2023-11-04

**Authors:** Jessica I. Wooden, Lauren E. Peacoe, Chinchusha Anasooya Shaji, Jennifer K. Melbourne, Cassie M. Chandler, Michael T. Bardo, Kimberly Nixon

**Affiliations:** 1 Division of Pharmacology and Toxicology, College of Pharmacy, The University of Texas at Austin, Austin, TX 78712, USA; 2 Department of Psychology, University of Kentucky, Lexington, KY 40506, USAmbardo@uky.edu (M.T.B.)

**Keywords:** adolescence, ethanol, microglia, astrocytes, social isolation, stress

## Abstract

During adolescence, the brain is highly susceptible to alcohol-induced damage and subsequent neuroimmune responses, effects which may enhance development of an alcohol use disorder (AUD). Neuroimmune reactions are implicated in adolescent alcohol exposure escalating adulthood drinking. Therefore, we investigated whether intermittent alcohol exposure in male, adolescent rats (AIE) escalated adult drinking via two-bottle choice (2BC). We also examined the influence of housing environment across three groups: standard (group-housed with enrichment during 2BC), impoverished (group-housed without enrichment during 2BC), or isolation (single-housed without bedding or enrichment throughout). In the standard group immediately after AIE/saline and after 2BC, we also examined the expression of microglial marker, Iba1, reactive astrocyte marker, vimentin, and neuronal cell death dye, FluoroJade B (FJB). We did not observe an escalation of adulthood drinking following AIE, regardless of housing condition. Further, only a modest neuroimmune response occurred after AIE in the standard group: no significant microglial reactivity or neuronal cell death was apparent using this model, although some astrocyte reactivity was detected in adolescence following AIE that resolved by adulthood. These data suggest that the lack of neuroimmune response in adolescence in this model may underlie the lack of escalation of alcohol drinking, which could not be modified through isolation stress.

## 1. Introduction

Adolescence, generally age 10–19+ [1], is a period when drug experimentation may initially occur, especially including the first exposure to alcohol. In the past month, over 15% of adolescents aged 12–20 years old report that they drank alcohol [2] while nearly 17% of 12th graders (age 17–18) “have been drunk” [3]. While adolescents may consume alcohol less frequently than adults, they drink more per occasion than adults, commonly in a binge pattern defined as 4+ or 5+ drinks per sitting for females and males, respectively [4,5]. Extreme binge drinking of 10–15 or more drinks in a sitting has also been on the rise [6,7]. Thus, adolescents drink to excess and in amounts that may have lasting consequences on their developing brains.

In humans, alcohol drinking in adolescence increases the likelihood of problematic alcohol use and diagnosis of AUD in adulthood [8,9,10,11,12]. Adolescence is a dynamic period of developmental plasticity in the brain that also corresponds with increased vulnerability to alcohol-induced effects that may drive development of an alcohol use disorder (AUD) [13,14,15,16]. Adolescents react distinctly to alcohol, including reduced sensitivity to its motor-impairing effects and enhanced sensitivity to its rewarding and positive effects [17,18]. These all predispose the adolescent to drink excessively, even though the adolescent is more vulnerable to alcohol-induced consequences [19,20].

One of the serious consequences of excessive alcohol consumption is its damaging effects on the nervous system. Specifically, alcohol damages corticolimbic regions of the brain and adolescents have greater damage from alcohol according to rodent models [19,20]. Part of the response to damage includes the activation of the neuroimmune system, especially microglia and astrocyte reactions [21,22,23,24,25,26,27,28,29,30]. Microglia, in particular, are the brain’s first responders to insult and neuroimmune effector, driving secondary toxicity and/or the resolution of damage depending on their phenotype [31,32]. While there is consensus that alcohol exposure causes a microglia reaction to some extent, whether the reaction is proinflammatory, reparative, or blunted and its precise role varies dramatically across studies (for discussion see [23]). Multiple studies performed in mouse models, especially C57Bl6J mice, suggest that alcohol-induced neuroimmune activation underlies the escalation of alcohol drinking [33,34] and microglia are specifically implicated [35]. 

Exposure to alcohol during adolescence also escalates adult drinking in some animal models [36,37,38,39,40,41] but not others, e.g., [42,43,44,45,46]. For example, in rats, escalation in drinking following adolescent exposure has been modeled with adolescent intermittent ethanol exposure (AIE) followed by testing two-bottle free-choice (2BC) alcohol drinking in adulthood [36,37,39,47]. However, both forced exposure, such as binge models via intragastric gavage or as sole source of liquids, and some voluntary consumption models, such as 2BC, have failed to produce an escalation in adult alcohol drinking [42,43,44,45,46]. One commonality among the models that successfully escalated adult drinking is single housing of the animals. Within the last 15 years there has been a shift to social housing with enrichment as the standard to improve the welfare of rodents [48,49], but in many of these older studies rats were singly housed. Further, rats are often single-housed during 2BC in order to precisely measure intake, although single housing can increase corticosterone levels [50] and increase rates of alcohol drinking and drug self-administration [51,52,53,54,55,56,57]. Environmental enrichment (EE) in the home cage of single-housed rodents allows them to experience a range of natural behaviors while removing enrichment and/or housing the rats in isolation conditions can increase the incidence of stereotypic and perseverative behaviors [58], as well as impair normal brain development and function (reviewed in [59,60,61]). 

Therefore, as there have been mixed results in the literature, and it remains unclear whether adolescent alcohol exposure is sufficient to drive these neuroimmune effects and subsequent drinking behavior, we conducted three experiments that manipulated housing condition and utilized AIE vs. adolescent intermittent saline (AIS) in male Sprague Dawley rats. We then measured voluntary ethanol drinking in adulthood using 2BC. Finally, to probe the relationship between AIE effects on adult alcohol drinking and neurodegeneration and neuroimmune reactivity, we examined microglial marker, Iba1, reactive astrocyte marker, vimentin, and neurodegeneration marker, FluoroJade B (FJB) in the rats in the standard group immediately after AIE or after adult drinking.

## 2. Methods

### 2.1. Animals and Housing Conditions

**Figure 1:**
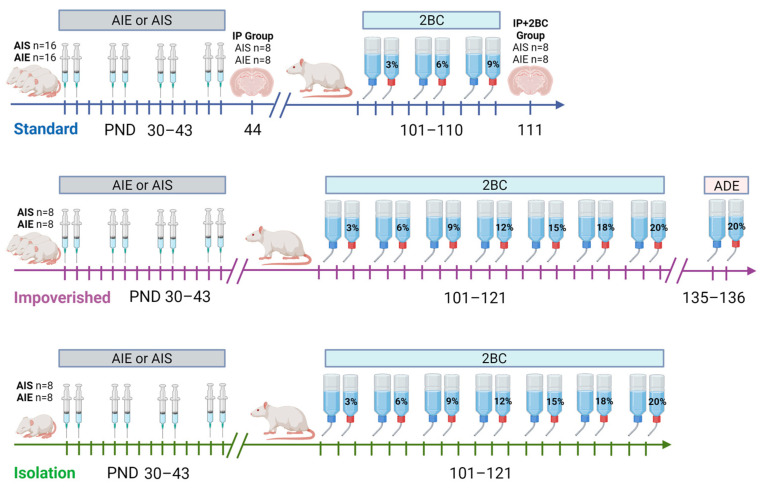
Timeline of experiments. Rats in the standard group were housed normally with environmental enrichment (EE), impoverished rats were housed without EE but otherwise normally, and rats in the isolation group were singly housed without EE, bedding, or visual access to other cages. All rats were singly housed during two-bottle choice (2BC). AIE = adolescent intermittent ethanol, AIS = adolescent intermittent saline, ADE = alcohol deprivation effect testing, IP group = examined in adolescence after AIE/AIS injections, IP+2BC group = examined in adulthood after AIS/AIE injections and 2BC. Image created with BioRender.com.

Adolescent male Sprague Dawley rats (*n* = 64) were purchased from Charles River Laboratories (Raleigh, NC, USA) and arrived at our facility at postnatal day (PND) 21. Male rats were utilized because our lab has previously shown that under a forced consumption paradigm, adolescent alcohol exposure impacted adult drinking in male rats only [43] and past experiments that we were attempting to replicate were conducted in males only [36]. All rats were acclimated to polycarbonate cages in an AAALAC-accredited vivarium at The University of Texas at Austin with a 12:12 h light/dark cycle and ad libitum access to rat chow and water for 7 days before experiments began. Rats were randomly assigned to either adolescent ethanol or saline groups upon arrival and subjected to one of three housing conditions: standard, impoverished, or isolation (see Figure 1). For the standard and impoverished groups, rats were group housed (2–3 per cage) while rats in the isolation condition were singly housed from arrival at our facility. Rats in the standard condition had access to NIH-guided environmental enrichment (EE, PVC pipe) in the home cage, while those in the impoverished and isolation conditions did not. Polycarbonate cages were used for all rats in the study, but rats in the isolation condition had metal grid flooring with no bedding material, and cages were visually blocked off from one another using poster board, to mimic social isolation and housing conditions as in our previous experiments [42,53,54]. All animal experimentation was approved in advance by The University of Texas at Austin Institutional Animal Care and Use Committee and was consistent with the Guide for the Care and Use of Laboratory Animals [49]. 

### 2.2. Adolescent Ethanol Exposure

For each housing condition, rats were further divided into adolescent ethanol or saline groups (Figure 1). Adolescent intermittent ethanol (AIE) consisted of 8 *i.p.* injections of 2 g/kg ethanol (20% *w*/*v*; Pharmco-Aaper, Shelbyville, KY, USA), and for adolescent intermittent saline (AIS) injections were 0.9% saline (Aspen Veterinary Resources, Liberty, MO, USA), in a 2 days on/2 days off manner from PND 30–43 [36,37,62]. At 1 h after the last ethanol injection, all AIE rats had tail blood obtained to verify blood ethanol concentration (BEC) during intoxication. Blood was collected via heparinized capillary tubes then transferred to microcentrifuge tubes containing heparin (3 µL, Meitheal Pharmaceuticals, Chicago, IL, USA). Samples were centrifuged at 6500× *g* rpm for 5 min, and plasma was collected and stored at −20 °C. Plasma was analyzed using an AM1 Alcohol Analyzer (Analox Instruments, Lunenburg, MA, USA) and samples were run in duplicate. At PND 44, half of the rats from the standard housing group were sacrificed via transcardial perfusion (IP group; AIE *n* = 8, AIS *n* = 8) and brain harvested to assess neuroimmune response and cell death immediately following AIE. On PND 101, free-choice alcohol drinking began for the remaining standard rats, as well as the impoverished and isolation rats. 

### 2.3. Alcohol Drinking in Adulthood

Alcohol drinking behavior in adulthood was examined via a 2-bottle free-choice drinking paradigm (2-bottle choice, 2BC). First, rats were acclimated to being singly housed with two bottles containing distilled water in the home cage (PND 87–100). On PND 101, one of the water bottles was switched out for ethanol in distilled water, while the other bottle always contained water (as previously described) [42]. Access to alcohol was not limited, and the two bottles were left in the cages 24 h a day. For the standard group, rats were offered increasing concentrations of ethanol every 3 days ranging from 3% to 9%, as described [36]. One day after cessation of 2BC (PND 111), rats in the standard group were transcardially perfused (2BC group; AIE *n* = 8, AIS *n* = 8) and brains harvested. For the impoverished and isolation groups, rats were offered an identical 3% to 9% range of ethanol concentrations beginning on PND 101, but then extended up to 20%, as used previously [42]. The two bottles in each cage were counterbalanced for left or right side daily, and bottles were weighed every day to measure intake. To account for bottle leakage, two empty cages were set up with a water bottle and an ethanol bottle inside, and the average fluid lost from the respective ethanol or water bottles was subtracted from intake when determining daily mL consumed. Rats normally will show an increase in alcohol consumption following a period of abstinence, known as the alcohol deprivation effect (ADE) [63]. In order to examine whether an ADE could be observed, rats in the impoverished condition were subjected to abstinence for 2 weeks (PND 121–135) and examined for the ADE: 2BC was returned with 20% ethanol for two days.

### 2.4. Histology

Rats from the standard housing group were used for histological studies at 1 day after the last ethanol injection (IP group, PND 44) or 1 day after 2BC ended (IP+2BC group, PND 111). Rats were deeply anesthetized with pentobarbital (Fatal Plus; Vortech Pharmaceuticals, Dearborn, MI, USA), checked for toe pinch and auditory reflexes to ensure anesthetic depth, and perfused transcardially with phosphate-buffered saline (PBS, pH 7.4) then 4% paraformaldehyde (pH 7.4; Acros Organics, Fair Lawn, NJ, USA). Brains were extracted, postfixed in 4% paraformaldehyde overnight then rinsed and stored in PBS at 4 °C until sectioning. Brains were sliced coronally into 40 μm sections in a 1:12 series using a vibrating microtome (Leica VT100S; Leica, Wetzlar, Germany) starting at approximately Bregma 5.64 mm through the caudal extent of the cerebrum [64]. Sections were preserved in cryoprotectant at −20 °C in 24-well plates until histological processing.

FluoroJade B (FJB; MilliporeSigma, Burlington, MA, USA) was chosen as it reliably detects degenerating neurons in our histological preparation, with better signal-to-noise than FluoroJade C [29,65,66,67]. To stain for FJB, every twelfth brain section was first mounted on positively charged Superfrost Plus^®^ slides (Fisher Scientific, Waltham, MA, USA) and allowed to dry thoroughly via slide warmer for 1 h, then overnight at room temperature. Slides were dipped in 1% NaOH in 80% EtOH for 5 min, 70% EtOH for 2 min, ddH_2_0 for 2 min, and 0.06% KMnO_4_ for 10 min, before being incubated in FJB in the dark for 20 min, exactly as previously described [29,68]. Following the 3 final ddH_2_0 washes, slides were then dried on a slide warmer and coverslipped using Cytoseal^®^ (Thermo Fisher Scientific, Waltham, MA, USA). A positive control section from a rat subjected to traumatic brain injury was processed simultaneously.

Free-floating immunohistochemistry (IHC) for Iba1 (microglia-specific; [69]) and vimentin (reactive astrocytes; [70]) was conducted on adjacent sections as previously described [27,29,66]. Vimentin is an intermediate filament protein expressed in reactive astrocytes [29,70]. For each antibody, adjacent sections were used: a series of every twelfth section was washed with tris-buffered saline (TBS; 3 × 10 min) and then incubated in 0.6% hydrogen peroxide. After washing with TBS, the tissue was placed in blocking buffer containing 0.1% Triton-X (Fisher Bioreagents, Fair Lawn, NJ, USA) in TBS and 3% goat serum (Vector Labs, Newark, CA, USA) for Iba1, or 3% horse serum (Vector Labs, Newark, CA, USA) for vimentin. The tissue was incubated in either 1:400 rabbit anti-Iba1 (Wako, Richmond, VA, USA) or 1:750 mouse anti-vimentin (Chemicon, Temecula, CA, USA) overnight at 4 °C. Dilutions were determined in pilot studies with negative controls where the primary antibody was omitted. After 24 h, sections were washed with blocking solution (3 × 10 min), then incubated in secondary antibody (1:200 goat anti-rabbit for Iba1, and 1:200 horse anti-mouse for vimentin; Vector Labs) for 1 h. Following washes in TBS, tissue was immersed for 1 h in avidin-biotin complex (Vectastain Elite ABC kit, Vector Labs, Burlingame, CA, USA), and then developed in nickel-enhanced 3,3′-Diaminobenzidine Tetrahydrochloride (DAB; Vector Labs, Burlingame, CA, USA). Sections were then washed in TBS, mounted in ddH_2_O, and coverslipped using Cytoseal^®^.

In order to verify that our vimentin antibody was indeed marking astrocytes, we assessed vimentin plus GFAP or Iba1 co-labeling using multilabel immunofluorescence and confocal microscopy. Glial fibrillary acidic protein (GFAP) reliably labels astrocytes in the rodent CNS [71]. Briefly, a few sections selected from brains from each group were rinsed in TBS then blocked in 10% goat serum/0.1% Triton-X/TBS. Tissue was then incubated overnight on a shaker at 4 °C in blocking solution with rabbit anti-GFAP (DAKO, Glostrup, Denmark, 1:2500) or rabbit anti-Iba1 (Wako, Richmond, VA, USA, 1:400) along with mouse anti-vimentin (Chemicon, Rolling Meadows, IL, USA, 1:400). Tissue was then rinsed 3 × 10 min in blocking solution and incubated in Alexa Fluor 555 anti-mouse (Invitrogen, Waltham, MA, USA, 1:200) and Alexa-Fluor 488 anti-rabbit (Invitrogen, 1:200). Slices were rinsed in TBS, mounted in ddH_2_O on glass slides, and coverslipped with ProLong Gold Anti-fade mounting medium (Invitrogen). To assess co-labeling of vimentin and GFAP or Iba1, z-stack images were taken with a 60× oil immersion lens (Olympus UPLXAPO) on an FV300 laser scanning confocal microscope (Olympus, Center Valley, PA, USA).

### 2.5. Quantification

Profile counting was used for FJB+ cells and vimentin+ cells in the hippocampus only. For FJB, profile counting was chosen due the paucity of cells and lack of background staining to define regions [67,72]. For vimentin+ cells in the hippocampus, profile counting allowed us to avoid counting progenitor cells in the dentate gyrus subgranular zone and focus on what appeared to be reactive astrocytes along the hippocampal fissure in the molecular layer of the dentate gyrus. After coding the slides to ensure blinding of the experimenter, FJB+ cells were manually counted on a BX-51 Olympus microscope (Olympus, Center Valley, PA, USA) under blue light excitation (488 nm). Brain sections were surveyed for FJB+ cells at a magnification that ranged from 10× to 80×, depending on the region being examined. When cells were observed, the accompanying location was identified using a rat brain atlas [64]. Data were recorded as cells per section, as justified previously [67]. For vimentin+ cell counts (hippocampus only), slides were coded and a light microscope (Olympus BX-43; Center Valley, PA, USA) was used to count vimentin+ cells in the dorsal hippocampus (approximately Bregma −1.80 through −4.2 mm). A 10× objective was used to survey the tissue, followed by 40× objective to verify cell number. Data are reported as mean vimentin+ cells per section.

Image analysis tools were used to assess Iba1 and vimentin immunoreactivity. For Iba1, we first assessed Iba1 immunoreactivity with densitometry. We chose to analyze regions sensitive to alcohol neurotoxicity: the perirhinal and entorhinal cortices (peri/ento-rhinal) and hippocampus. Slides were coded and imaged on a light microscope (Olympus BX-43). In ImageJ (Fiji version 2.9.0), images were pre-processed: sharpen and unsharp mask were applied, followed by pseudo flat field correction (BioVoxxel Plugin). A threshold of 10:200 was used to record the % immunoreactive area in each of 3 images taken in each region. For the hippocampus, images were taken at approximately Bregma −2.92, −3.36, and −4.08 mm, while in the entorhinal cortex images were taken at Bregma −4.08, −4.8, and −5.76 mm. In the hippocampus and peri-/entorhinal cortex, microglia were also assessed for soma size differences between groups. Using a macro workflow in ImageJ (Fiji), images of Iba1 stained tissue in the hippocampus and entorhinal cortex were pre-processed to reduce background noise, thresholded to capture only the cell soma, then particles were analyzed for average object area. For vimentin, the brain was surveyed and two regions with visibly distinct vimentin + immunoreactivity were selected for quantification: the hippocampus (profile counts above) and the forceps minor of the corpus callosum (CC_fm_). Past work has shown that adolescents have damage in these two regions in particular [73,74,75]. In sections where the CC_fm_ was visible, three images were taken at 40×: Box A placed proximally, Box B placed dorsally, and Box C placed laterally. Percent area values were averaged across the three boxes and reported as mean percent area ± SEM. 

### 2.6. Statistical Approaches

All data were assembled in Microsoft Excel and analyzed using Graphpad Prism 10 (GraphPad Software, La Jolla, CA, USA). BEC and subject body weight were analyzed by one-way ANOVA, except for body weight across adolescence which was analyzed using repeated measures ANOVA. Alcohol drinking data was analyzed by either one-way ANOVA, mixed-model ANOVA, or repeated measures ANOVA. Two-way ANOVA was used for all cell quantifications, and Bonferroni post hoc tests were employed as was appropriate. All data are reported as mean ± standard error of the mean (SEM). Statistical significance was accepted at *p* < 0.05. 

## 3. Results

### 3.1. Social Isolation Increased Body Weight

**Figure 2:**
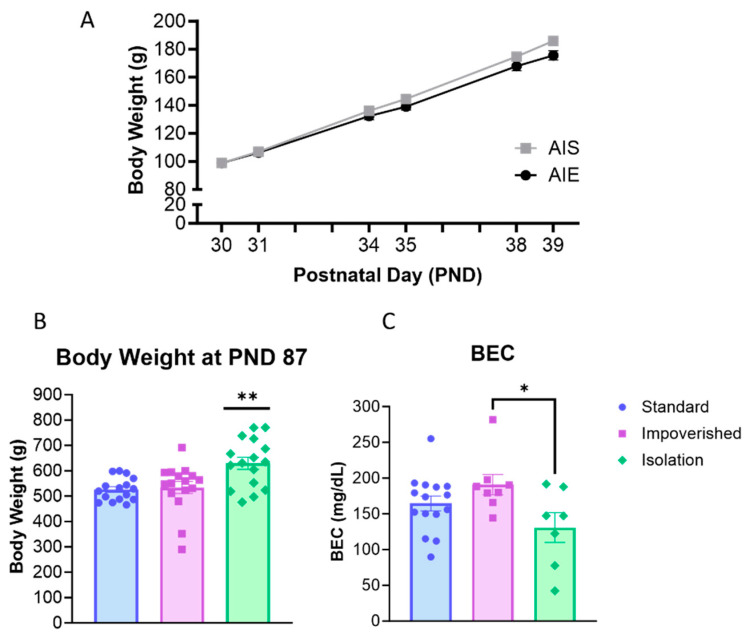
Body weight and blood ethanol concentration (BEC). (**A**) All rats gained weight across adolescence during adolescent intermittent ethanol exposure (AIE). (**B**) In adulthood, body weight was significantly higher in the isolation group compared to the standard or impoverished groups. (**C**) BEC was slightly lower in the isolation group compared to impoverished. * *p* < 0.05, ** *p* < 0.01.

AIE rats were injected with 2 g/kg ethanol for two days on/two days off from PND 30–43 according to previous work [36]. Collapsed across the three housing manipulations (experiments), rats weighed 98.9 ± 1.6 g at PND 30 and gained weight across AIE/AIS treatment (Figure 2A). For body weight across adolescence, repeated measures ANOVA shows a main effect of time [F(2.312, 141.0) = 3329, *p* < 0.0001], as well as a significant interaction for AIE X Time [F(5, 305) = 12.77, *p* < 0.0001], but no significant effect of AIE alone. 

When 2BC began in adulthood (PND 101), there was no difference between AIE and AIS rats for body weight. However, one-way ANOVA shows that body weight differed between experiment groups [F(2, 45) = 7.775, *p* = 0.0013], with Bonferroni post hoc comparison indicating significantly higher body weight in the isolation group compared to impoverished (*p* = 0.0062) or standard (*p* = 0.0028; Figure 2B). At 1 h after the last injection, BEC averaged 158.8 ± 16.3 mg/dL for all rats and was significantly different across experiments [F(2, 27) = 3.480, *p* = 0.0452]: 154.6 ± 13.9 mg/dL for the standard group, 190.9 ± 14.2 mg/dL for the impoverished group, and 130.9 ± 20.8 for the isolation group (Figure 2C). 

### 3.2. AIE Did Not Alter Adult Alcohol Drinking, with or without Social Isolation

**Figure 3:**
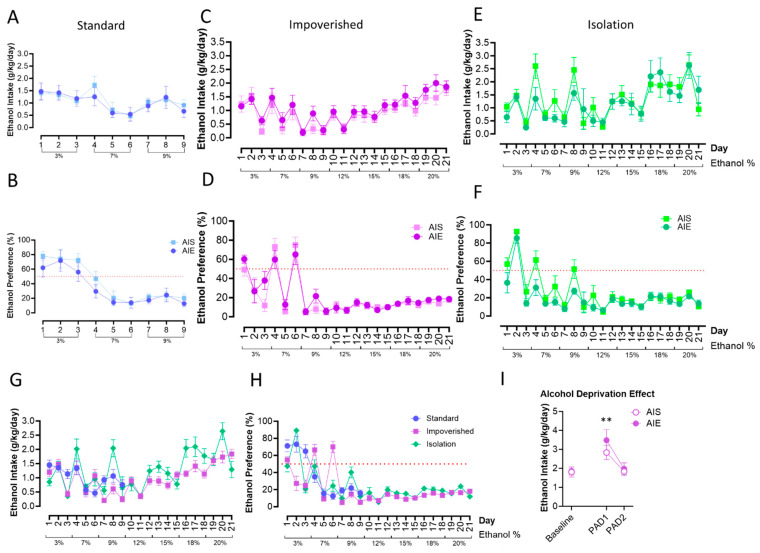
Two-bottle choice (2BC) drinking in adulthood. AIE did not influence ethanol intake or preference in the standard groups (**A**,**B**), the impoverished groups (**C**,**D**), or the isolation groups (**E**,**F**). Ethanol intake (**G**) and preference (**H**) were similar across experiments, shown here collapsed across AIE/AIS groups. Rats in the impoverished group demonstrated increased alcohol consumption following a period of deprivation, the “alcohol deprivation effect”, at the first day post-abstinence (PAD1), but intake returned to baseline by PAD2 (**I**). *n* = 7–8/group ** *p* < 0.01.

Free-choice drinking under 2BC began on PND 101. For the standard housing rats, repeated measures ANOVA revealed that there were no significant changes to ethanol intake or preference as a result of AIE (Figure 3A,B). However, there was a significant effect of time [F(3.513, 45.66) = 4.557, *p* = 0.0049]: intake generally decreased over time, as alcohol percentage went up. Similarly, there was no effect of AIE in the impoverished groups (Figure 3C,D), although there was a main effect of time for intake [F(2.773, 36.05) = 9.823, *p* = 0.0001]. In the isolation groups (Figure 3E,F) there was a main effect of time [F(5.076, 65.74) = 10.06, *p* < 0.0001] as well.

Finally, we compared alcohol intake and preference between the housing groups only. Mixed-model ANOVA did not show a difference between housing groups for ethanol consumption or preference, although there was a significant effect of time [F(7.038, 253.0) = 16.91, *p* < 0.0001; Figure 3G,H]. In the impoverished group, we also tested whether an alcohol deprivation effect (ADE; an increase in alcohol consumption after a period of deprivation; [63]) would be observed or differ between groups. In the impoverished group, both AIE and AIS rats displayed increased intake of 20% EtOH during day one post-abstinence (PAD1) compared to baseline [t(7) = 3.610, *p* = 0.0086 for AIE, t(7) = 4.236, *p* = 0.0039 for AIS], although consumption was similar between the two groups. Drinking returned to baseline levels by PAD2 (Figure 3I).

### 3.3. Neuronal Cell Death Was Not Modified by AIE or Adult Alcohol Consumption

**Figure 4:**
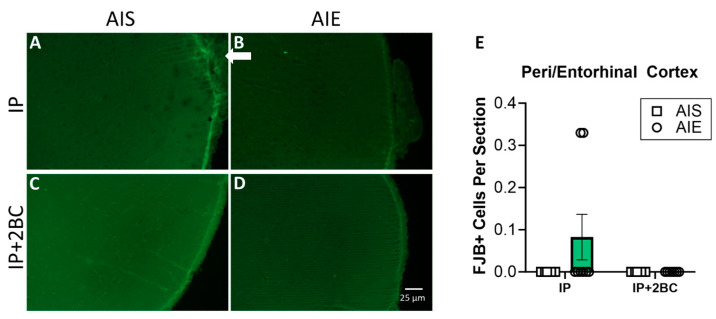
FluoroJade B (FJB) staining and quantification in the IP group (PND 44) and the IP+2BC group (PND 111). Representative images of FJB staining in the entorhinal cortex (**A**–**D**). A few FJB+ cells were detected in the peri-/entorhinal cortex of a few rats (arrow in (**B**)) as quantified in (**E**), but the difference between groups was not significant. *n* = 7–8/group.

Brains were surveyed extensively for FJB+ cells by an observer blind to treatment groups. The peri-/entorhinal cortices showed distinguishable FJB+ cells, so this region was chosen for specific quantification. No FJB+ cells were detected in the hippocampus of any rat. FJB staining is shown in the hippocampus (Supplemental Figure S1) and peri-/entorhinal cortices (Figure 4A–D). Two-way ANOVA did not show a significant difference between groups for the peri-/entorhinal cortex (Figure 4E). 

### 3.4. AIE Does Not Enhance Microglia Reactivity

**Figure 5:**
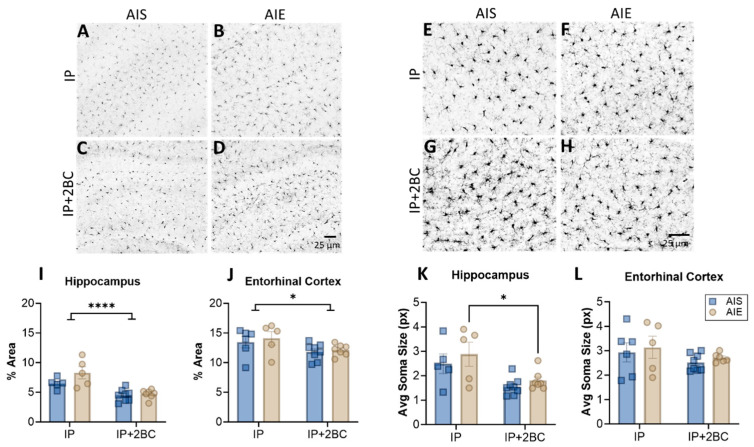
Microglia in the hippocampus (**A**–**D**) and entorhinal cortex (**E**–**H**). Iba1 immunoreactivity was higher in the IP groups compared to the IP+2BC groups for both regions (**I**,**J**). In the hippocampus, microglia soma size was decreased in the IP+2BC group compared to IP, for AIE rats (**K**). Soma size in the entorhinal cortex did not differ between groups (**L**). *n* = 5–8/group, * *p* < 0.05, **** *p* < 0.0001.

Brain sections were examined for Iba1 immunoreactivity and microglia soma size in the hippocampus (Figure 5A–D) and entorhinal cortex (Figure 5E–H) in the IP group (PND 44) and the IP+2BC group (PND 111). In the hippocampus, two-way ANOVA revealed a main effect of treatment [IP vs. IP+2BC: F(1, 21) = 27.77, *p* < 0.0001] but no interaction or effect of AIE alone (Figure 5I). In the entorhinal cortex, there was also only a main effect of treatment [F(1, 22) = 6.074, *p* = 0.022; Figure 5J]. In both regions lower Iba1 density is apparent in the 2BC group compared to the IP group, perhaps a result of the age of the rats after 2BC. Similarly, in the hippocampus, there was decreased microglia soma size in the IP+2BC group compared to IP [F(1, 21) = 12.82, *p* = 0.0018], with AIE rats in particular driving this effect (*p* = 0.0283; Figure 5K). However, in the entorhinal cortex, soma size did not vary by group (Figure 5L).

### 3.5. AIE Increased Astrocyte Reactivity in Adolescence, but Not Following Adult Alcohol Drinking

**Figure 6:**
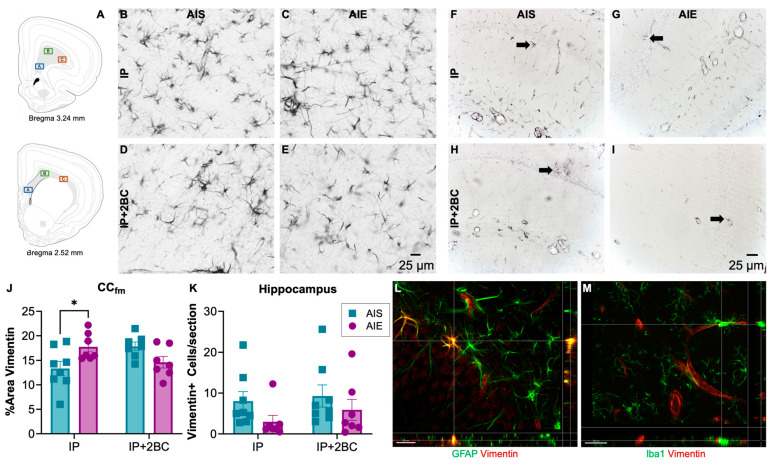
Vimentin immunoreactivity. Approximate location of images taken in the forceps minor of the corpus callosum (CC_fm,_ (**A**)). Image adapted from [76]. Representative images of vimentin immunoreactivity in the CC_fm_ (**B**–**E**) and the hippocampus (**F**–**I**). In the CC_fm_, AIE rats had a 33 ± 0.02% increase in astrocyte reactivity following *i.p.* injection that resolved even after 2BC drinking (**J**). When quantifying vimentin+ cells in the hippocampus, there were fewer cells counted in AIE rats regardless of treatment condition, but this effect was not statistically significant (**K**). Fluorescent double-labelled images verifying that vimentin+ immunoreactivity (red) co-localized with astrocyte marker, GFAP (green) (**L**) but not with microglia marker Iba1 (green) (**M**) in the hippocampus. *n* = 7–8/group, * *p* < 0.05.

Brains were surveyed for vimentin immunoreactivity (vim+IR) in the IP group (PND 44) and in the IP+2BC group (PND 111). The hippocampus and forceps minor of the corpus callosum were chosen for quantification due to prior reports of damage in these regions [73,74,75]. In the corpus callosum (Figure 6A,B–E), densitometry was performed and two-way ANOVA revealed no main effects, though a significant interaction [F(1, 25) = 10.30, *p* = 0.0036], reflected that AIE rats had a 33 ± 0.02% increase in vim+IR (*p* = 0.03) following *i.p.* injection. However, after 2BC, there was no longer a difference between AIS and AIE rats (Figure 6J). 

Profile counts were obtained for vimentin+ cells in the dorsal hippocampus (Figure 6F–I). Two-way ANOVA revealed a trend (*p* = 0.0877) for a main effect of AIE but no significant interaction with treatment condition (IP vs. IP+2BC). Thus, slightly fewer vimentin+ cells (n.s.) were counted in the alcohol groups regardless of treatment condition (Figure 6K). 

## 4. Discussion

AIE exposure utilizing *i.p.* injections was used in the current study as a model of binge-like exposure to alcohol during adolescence. Although adolescents do not drink as often as adults, they drink in larger quantities when they do drink, consistent with their high rates of binge drinking [5]. Indeed, 8.3% of adolescents report binge drinking in the last 30 days [2] while a minority drink in a dangerous pattern of high intensity or extreme binge drinking of 15 or more drinks in a sitting [6,77]. The adolescent’s reduced sensitivity to factors that help one control their alcohol intake such as diminished motor impairing or sedative effects of alcohol, coupled with their greater vulnerability to the damaging effects of alcohol, e.g., [19], sets up a dangerous situation [17]. Unfortunately, excessive alcohol exposure in adolescence changes the brain in such a way as to increase the likelihood that these individuals develop an AUD as adults [9,10,11,12]. 

Fundamental to alcohol problems in adulthood is increased consumption, the effect we hoped to model here. We previously observed no escalation in alcohol drinking after binge-like ethanol in both 4-day (unpublished observations) and 2-day exposure via intragastric gavage [43] as well as with voluntary consumption via two-bottle choice [42]. Therefore, we aimed to replicate a model of adolescent alcohol exposure that was documented to escalate adult drinking: a 2 g/kg dose was administered *i.p.* intermittently (2 days on/2 days off) across PND 30 to 43 in male Sprague Dawley rats [36,37]. When we did not see an escalation in adulthood drinking following AIE using the standard housing approach (group housing except during 2BC, where rats were provided a PVC pipe for enrichment), we dug deeper into the details of housing and environment on alcohol drinking behavior [78]. Next, we used an impoverished condition, where the rats were group-housed upon arrival but had no environmental enrichment (EE) during the single housing necessary for 2BC as adults. Unfortunately, the AIE rats in the impoverished condition still did not escalate their drinking above the level of the saline controls. Finally, as the impoverished condition only had one source of stress (lack of EE), we employed an isolation condition to induce a more stressful environment [53,54] where the rats were singly housed without bedding or enrichment from adolescence. For the isolation condition, the cages were also visually blocked off from one another, to mimic the hanging metal cages used when we a observed a slight escalation in sweetened alcohol drinking previously [42]. As can be seen in Figure 3E,F, isolation did not interact with adolescent alcohol exposure to escalate adult alcohol drinking by 2BC. Evaluating each of these alcohol groups together (Figure 3G,H) might suggest visually that there was a slight increase in alcohol drinking in the isolation group compared to the other two housing groups, but the AIE-isolation rats looked similar to their saline-injected counterparts (Figure 3E,F). We acknowledge that even in our most severe isolation condition without bedding (metal floor grates) or visual cues, that our newer animal facility may not be sufficiently stressful. Thus, in summary, contrary to our hypothesis, none of the environmental manipulations had a significant impact on alcohol drinking via 2BC in adulthood.

Although rats in the standard and impoverished housing conditions were group-housed in adolescence and adulthood prior to 2BC, it should be noted that all rats in all housing conditions were subjected to isolate housing in preparation for and during 2BC (with isolate housing lasting between 24 and 49 days). This is commonly carried out in order to precisely measure the amount of intake for each rat, but as we acknowledge that isolate housing during adolescence could influence drinking behavior, we also recognize that this period of isolation during adulthood free-choice drinking could have influenced behavior for all rats in the study. However, levels of alcohol drinking in the current study were similar to those found in previous studies using a continuous access model in male Sprague Dawley rats [37]. Additionally, BEC in the isolation housing group was slightly lower than in the standard or impoverished groups. While this could be perceived as a potential confound, the low number is driven by one particularly low value. BECs are merely a snapshot of the exposure on that single day and can vary [79]. Furthermore, previous studies using the AIE-2BC model either did not report BEC or did not report BEC values for rats in the continuous access group, making it difficult to compare between studies or determine if adolescent BEC differences contributed to the significant differences between groups [36,37]. Thus, this slight difference in BEC does not appear to underlie our lack of effect.

Thus, these data add to the growing collection of reports that were unable to replicate the few studies where rats exposed to alcohol in adolescence escalate their voluntary consumption of alcohol in adulthood. A few groups report increased drinking following ethanol exposure during adolescence in rats [36,37,38,39,41,80] even through late adolescence [40] and in both sexes using a variety of adolescent alcohol exposure paradigms including the *i.p.* injections used here [81]. Similar results can be found in mouse models as well, with increased intake or preference in adulthood [82,83], although this effect appears to be dependent on sex [84] and strain (increase in C57BL/6J mice but not in the DBA2/J strain, see discussion below [85]). However, other laboratories find no change or even a decrease in adulthood drinking after adolescent ethanol in rats [45,46], and mice (no increase in DBA2/J mice [85]). Notably, decreased adult consumption was seen in another study using an adolescent *i.p.* injection model [44]. Similarly, also following a forced binge-like ethanol exposure via intragastric gavage in rats, we do not see increased drinking in adulthood, and may even see evidence of an aversive effect [42,43].

Failure to replicate previous studies’ findings can occur for several reasons. These differences may be due to methodological details that can be controlled (strain, sex, dose, housing, etc.) but importantly, ones that cannot (e.g., [86]). While we matched the strain, sex, and dose, there were some differences in the source of the rats with ours using Sprague Dawley weanlings from Charles River and others studies report in-house colonies [37] or pregnant dams from Harlan [36]. Furthermore, while some had success with Wistars [39], others did not [44]. Thus, as a variety of rats and sources have been successful or not, we rule this out as a major factor. Perhaps the most striking reasons that are far from control are the differences between laboratory facilities, even including animal delivery methods and experimenters [86,87]. Modern housing facilities are often equipped with lighting, ventilation, and noise insulation that are more comfortable for rodents, although buildings constructed before directives for such measures existed probably did not take these parameters into consideration [48,49]. Additionally, the ability to mitigate potential stressors for rodents varies between locations, such as altering the intensity and frequency of overhead lighting, reverse light/dark cycles, noise levels, ventilation systems that reduce ammonia buildup in cages, frequency and manner of bedding changes, male vs. female experimenters [88], using environmental enrichment and group housing as the standard [48,49], and so on, all of which can alter stress responses in rats (reviewed in [89]). For example, UT Austin animal facilities no longer utilize the hanging-rack metal cages that were used previously [42]. Thus, in the current experiment, we modified polycarbonate cages to have a metal grid floor and white poster board between the cages to best mimic the isolation experience of the hanging metal cages. However, the polycarbonate cages have nearly twice the amount of space and provide better lighting inside the cage, which is a distinctly different experience. Furthermore, our animal facility is a small satellite facility that is relatively new with few animals, is generally quiet, and has been evaluated to ensure lighting and HVAC systems are ultrasonically quiet. Perhaps our animal environment is very different from these past reports [86] and just the right combination of potential stressors must be introduced to see drinking effects in adulthood.

Recent work has shown that the escalation of alcohol drinking is related to neuroimmune activation and specifically microglia reactions in adult alcohol models, though adolescent rodents have yet to be studied [34,35,90]. The neuroimmune hypothesis of AUD development supports that alcohol effects on microglia (or astrocytes) may underlie the downward spiral into addiction [33,91,92,93,94]. Multiple studies indicate that this hypothesis may especially apply to adolescents [13,95,96]. We and others have shown that alcohol causes a microglial reaction in adolescence [23,28,95,97,98,99]. However, whether the reaction is proinflammatory, reparative, or blunted, and its precise role varies dramatically across studies (for critical review see [23]). For example, studies performed in mouse models, especially C57BL/6J mice, suggest that alcohol-induced neuroimmune activation underlies the escalation of alcohol drinking [94,100] and microglia are specifically implicated [35]. Accordingly, C57BL/6J mice, which demonstrate a greater proinflammatory response to alcohol than rats and other mouse strains [101], escalate their drinking after adolescent exposure [80,82,85,102]. However, a caveat is that the C57BL/6J’s heightened inflammatory response is likely due to their mutation in the *nnt* gene that renders them at a higher pro-oxidant baseline level and thus more sensitive to alcohol damage in development [103,104]. Indeed, while C57BL/6J mice will escalate their alcohol drinking in adulthood after adolescent exposure, DBA/2J mice, without the mutated *nnt* gene, do not [85]. Rats also do not show the dramatic inflammatory effects of alcohol but instead may have more reparative types of microglia that help to resolve damage or even a blunted response [28,105,106,107]; for review see [23]. Here, our histological analyses support that AIE with 2 g/kg ethanol does not cause a proinflammatory microglia reaction (Figure 5) or much of an astroglial reaction (Figure 6). Specifically, upregulation of the microglia-specific marker Iba1 [69] is not as dramatic as that observed in binge exposure models [28,108], nor does the morphology suggest that the microglia are proinflammatory [109,110]. We also did not observe significant FJB labeling of degenerating cells. While the lack of observable cell death theoretically could be due to the time at which we assessed FJB, 14 days after the first alcohol exposure when cellular debris may already be cleared [19,65], there is limited evidence to support that the relatively low BECs characteristic of either AIE with 2 g/kg ethanol or 2BC in these short timeframes would produce significant neurodegeneration. In agreement, FluoroJade dyes have not revealed cell death in either 2 g/kg IP ethanol administered for multiple days [111], drinking in the dark models [112] nor even with high BEC, acute doses through a full day of binge-like exposure [19,113,114]. Thus, the lack of inflammatory reaction in this particular AIE model may explain why rats did not escalate the drinking in adulthood. 

## 5. Conclusions

In summary, intermittent exposure to 2.0 g/kg of ethanol in adolescence did not produce an escalation in adult drinking in male rats, despite varying levels of isolation stress. Additionally, this adolescent *i.p.* exposure model did not produce significant neurodegeneration or microglial reactivity. In agreement, only modest changes in astrocyte reactivity were observed in adolescence, and not at all in adulthood, even after a second alcohol exposure via 2BC drinking. These results suggest that the lack of neuroinflammatory effect in adolescence underlies the lack of escalation in alcohol drinking in adulthood. In rodent models that are more predisposed to the inflammatory effects of ethanol, such as the C57BL/6J mouse, this escalation has been more consistently observed [80,82,85,102]. This may come down to the lack of classic pro-inflammatory neuroimmune effect in response to alcohol seen in rats as opposed to C57BL/6J mice [23], as well as the lack of additional stressors in our animal housing environment. 

## Data Availability

Data will be made publicly available according to NIH guidelines at: https://dataverse.tdl.org/dataverse/utexas.

## Supplementary Materials

The following supporting information can be downloaded at: https://www.mdpi.com/article/10.3390/cells12212572/s1, Figure S1: FluoroJade B (FJB). No FJB+ staining was found in the hippocampus of any rat (A–D). Positive control tissue subjected to TBI (E).
