# Supplementary material for: Adolescent Intermittent Ethanol Drives Modest Neuroinflammation but Does Not Escalate Drinking in Male Rats
Source: Cells. 2023 Nov 4;12(21):2572. doi: 10.3390/cells12212572 (PMC10649200; doi:10.3390/cells12212572)
Supplement: Supplementary file 1 [file cells-12-02572-s001.zip › cells-2615858-supplementary.pdf]

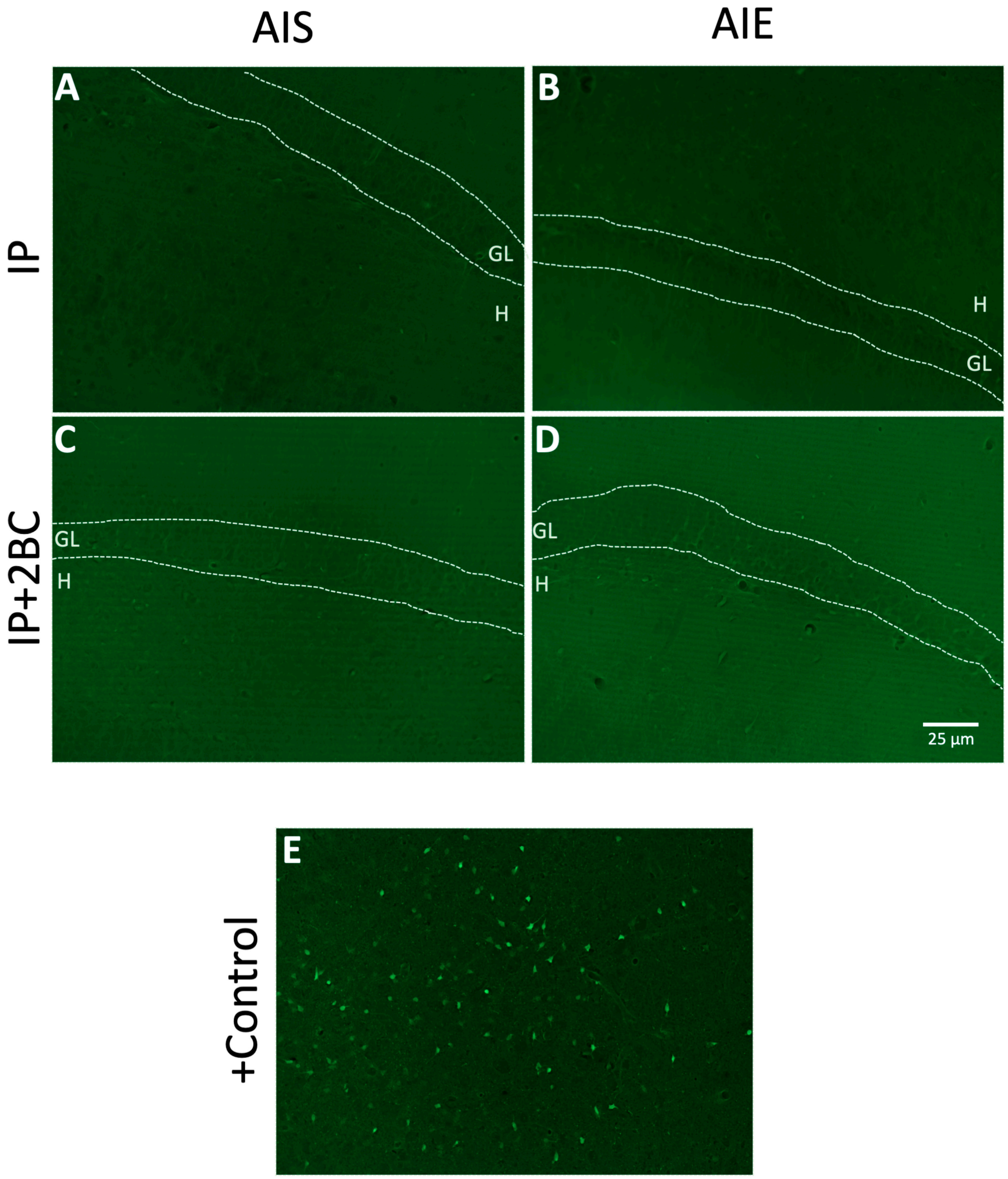

Figure S1. FluoroJade B (FJB). No FJB+ staining was found in the hippocampus of any rat (A-D). Positive control tissue subjected to TBI (E).
